# Non-contact mechanical and chemical analysis of single living cells by microspectroscopic techniques

**DOI:** 10.1038/lsa.2017.139

**Published:** 2018-02-09

**Authors:** Sara Mattana, Maurizio Mattarelli, Lorena Urbanelli, Krizia Sagini, Carla Emiliani, Mauro Dalla Serra, Daniele Fioretto, Silvia Caponi

**Affiliations:** 1 Department of Physics and Geology, University of Perugia, Perugia I-06123, Italy; 2 Laboratory of Biochemistry and Molecular Biology, Department of Chemistry, Biology and Biotechnology, University of Perugia, via del Giochetto, Perugia I-06123, Italy; 3 CEMIN-Center of Excellence for Innovative Nanostructured Material, Perugia I-06123, Italy; 4 Istituto di Biofisica CNR (IBF-CNR), Unità di Trento, and FBK, Via Sommarive 18, Trento 38123, Italy; 5 Istituto Officina dei Materiali del CNR (CNR-IOM)—Unità di Perugia, c/o Department of Physics and Geology, University of Perugia, Perugia I-06123, Italy

**Keywords:** living single cell analysis, mechanical properties, Raman and Brillouin spectroscopy

## Abstract

Innovative label-free microspectroscopy, which can simultaneously collect Brillouin and Raman signals, is used to characterize the viscoelastic properties and chemical composition of living cells with sub-micrometric resolution. The unprecedented statistical accuracy of the data combined with the high-frequency resolution and the high contrast of the recently built experimental setup permits the study of single living cells immersed in their buffer solution by contactless measurements. The Brillouin signal is deconvoluted in the buffer and the cell components, thereby revealing the mechanical heterogeneity inside the cell. In particular, a 20% increase is observed in the elastic modulus passing from the plasmatic membrane to the nucleus as distinguished by comparison with the Raman spectroscopic marker. Brillouin line shape analysis is even more relevant for the comparison of cells under physiological and pathological conditions. Following oncogene expression, cells show an overall reduction in the elastic modulus (15%) and apparent viscosity (50%). In a proof-of-principle experiment, the ability of this spectroscopic technique to characterize subcellular compartments and distinguish cell status was successfully tested. The results strongly support the future application of this technique for fundamental issues in the biomedical field.

## Introduction

In the vast research area of cellular biology, a largely followed approach consists in attempting to connect cell function with its complex architectural structure and the intricate hierarchy of its dynamic processes. However, cellular complexity prevents any single technique or experiment from revealing the details of the structure and active processes, which can span several decades in length and time. A multidisciplinary approach and the combined use of complementary techniques is an essential requirement to provide novel insights in this field. Bio-photonics and light-based technologies working in a contact-less configuration have been increasingly utilized to gain comprehension of fundamental biological issues. Thanks to recent improvements, confocal laser scanning microscopy, immunohistochemistry and fluorescence techniques^1, 2, 3, 4, 5^ allow for the acquisition of functional images that are pivotal for the characterization of the processes and distribution of molecular species in cells and also overcome the diffraction limit. Despite their impact, these approaches require chromophores and/or fluorescent dyes that selectively bind subcellular components and could ultimately interfere with the natural biological structures and processes under examination. A preferable label-free approach is offered by spectroscopic methods, such as Raman and Brillouin, based on inelastic light scattering processes. These two techniques provide complementary information. The characteristic Raman vibrational frequencies in the THz region provide information at the molecular level about the chemical composition and the structural molecular arrangement. Recently, the potential of the Raman approach has been clearly verified in *in vitro* cell analyses^6, 7^, as well as in medical applications^8^ that anticipate its future disruptive impact. For example, intraoperative tests have demonstrated unambiguous spectroscopic recognition of the cancer cell distribution in brain tissues^9^. More recently, Brillouin spectroscopy has been proposed as a novel imaging technique with potential application for the mechanical characterization of cells and tissues^10, 11, 12, 13, 14, 15, 16^. Based on the interaction of light with spontaneous acoustic phonons in the GHz frequency range, Brillouin spectroscopy is sensitive to the viscoelastic properties of the materials that probe the collective dynamics mediated over the acoustic phonon wavelength.

For many years the complementarity of these two techniques has been well exploited in material science and condensed matter physics to characterize both the structural and dynamical properties of a large variety of samples^17, 18, 19, 20^.

To analyze biomaterials that are intrinsically spatially heterogeneous and often affected by time dependent processes, the key requirements are *in situ* analysis and the simultaneity of the investigation. These strict requirements are now accessible by a recent evolution in the experimental setup which permits the light coming from a single scattering volume to be analyzed simultaneously by two different spectrometers^21, 22, 23^.

Extending the single Brillouin analysis^11, 13^, we propose the use of such combined spectroscopic approach to study the mechanical properties correlated with the biochemical composition of living fibroblasts (i) in label-free conditions, (ii) in a non-invasive manner and (iii) under physiological conditions. An interesting attempt in this direction was recently presented on erythrocytes^24^ which, lacking the cell nucleus and most organelles, are considered one of the simplest cell models.

The results presented in this work significantly expand upon previous investigations by analyzing complex and structured cells that may also undergo oncogenic transformation. Thanks to the unprecedented contrast and the unique frequency resolution of the newly developed homemade Brillouin spectrometer, we were able to analyze in detail not only the position but also the shape of the Brillouin peaks to provide a complete viscoelastic characterization. In particular, we found a mechanical modulation inside the cell that strongly correlates with the biochemical composition: a key role for proteins that aggregate into complex structures to form the cytoskeleton is clearly identified in this work.

Moreover, the ability of our technique to demonstrate small modifications of the elastic properties was successfully exploited, revealing a clear transition in both the elastic modulus and apparent viscosity following oncogenic expression. Our results demonstrate how the proposed method is not only of great interest for the analysis of fundamental biophysics, but also represents a strategic tool for future diagnostic studies.

## Materials and methods

### Experimental setup

**Figure 1:**
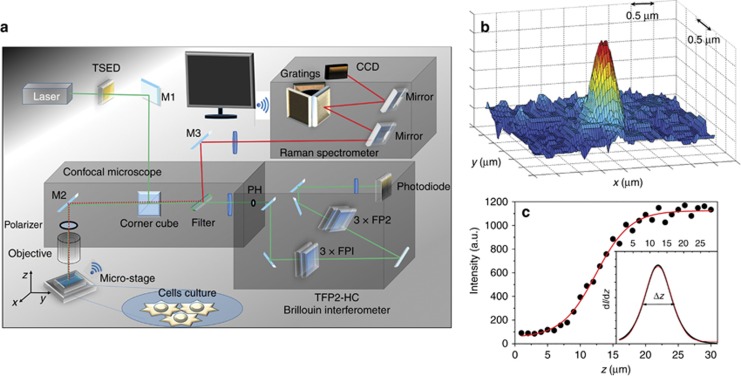
(**a**) Layout of the setup. The laser beam, once cleaned by the temperature stabilized etalon device (TSED), is focused onto the sample by the same microscope objective used to collect the backscattered light. Thanks to the three-axis piezo translation stage, the sample can be moved with a spatial resolution of ~0.01 μm. A polarizer can be used to select the polarization of the backscattered light and a short-pass tunable edge filter transmits the quasi-elastic scattered light (green beam) to the Brillouin spectrometer (TFP-2 HC) and reflects the inelastic scattered light (red beam) towards a Raman monochromator (a Triax Jobin-Ivon). (**b**) Measure of the lateral spatial resolution in the *x*–*y* plane. The light image has a Gaussian shape in both directions with a full width at half maximum Δ*x*=Δ*y*=(0.50±0.01) μm. (**c**) Edge spread function of the Brillouin peak intensity (I) of a vitreous silica slab acquired while crossing the interface with water. Inset: the derivative dI/dz has a full width at half maximum Δ*z*=(8.0±0.5) μm, providing the depth resolution of the experimental setup.

Figure 1a shows the layout of the experimental setup for the simultaneous Brillouin-Raman characterization. A single mode diode-pumped-solid state Spectra-Physics Excelsior laser operating at *λ*=532 nm was employed. The laser beam passes through the temperature controlled etalon, (TSED) TCF-1 from JRS Scientific Instruments (Tablestable Ltd., Mettmenstetten, Switzerland), which is designed to reduce the intensity of spurious secondary laser modes that lie in the region of interest for Brillouin measurements. Once filtered by neutral density filters, the laser beam is focalized on the sample using a customized CM-1 confocal microscope from JRS Scientific Instruments with a coaxial LED illuminator used to illuminate the sample surface and to select the investigation zone.

An infinity-corrected apochromatic water immersion objective UPLSAPO 60XW from Olympus was used both to focalize and to collect the back-scattered light. It is capable of capturing high quality images and intense spectra thanks to its high numerical aperture (NA=1.2). The excitation source has a power less than 3.5 mW and is focused onto a single cell through the objective, which is directly immersed into the cellular buffer solution. The collected scattered intensity is selected according to its frequency by a tunable ultrasteep short-pass filter (TEF, Semrock SP01-561RU), which transmits the anti-Stokes quasi-elastic scattered light to a Tandem Fabry Perot interferometer (TFP-2 HC) while it reflects the Stokes inelastically scattered light towards the Raman Spectrometer (RM- Horiba iHR320 Triax). To improve the rejection of the elastic contribution on the Raman spectra, a RazorEdge ultrasteep longpass edge filter (LPF LP02561RE—Semrock) was also used. The Raman spectra presented herein were acquired using the 600 grooves mm^−1^ grating and an N_2_ cooled CCD detector (1024 × 256 pixels) allowing the simultaneous acquisition of a 3000 cm^−1^ Raman shift in a single spectrum with a resolution of ~10 cm^−1^. The acquisition time for the whole spectrum is 300 s. The same time is used to acquire the Brillouin spectra through a TFP-2 HC, the high contrast variant of the original Sandercock type tandem multi-pass interferometer^25^.

The final spectral resolution of the Brillouin microscope is described in the details in the data analysis section. The in-plane spatial resolution of the optical arrangement is 0.5 μm × 0.5 μm, measured on the *x*–*y* surface by the laser spot diameter (Figure 1b). The spatial resolution along the *z* axis, parallel to the laser beam, is ~8 μm. It was measured, by exploiting the linear dependence of the Brillouin signal on the excitation power, from the edge spread function (ESF) of the Brillouin peak intensity of a reference flat sample (a transparent silica slab) acquired while crossing the interface with a different, equally transparent medium (water). The resolution was estimated as the FWHM of the line spread function obtained by the first derivative of the ESF^26, 27, 28, 29^. This value could be further reduced by decreasing the size of the entrance pinhole (PH) to the spectrometer at the cost of increasing the acquisition time. In fact, given our optical components, in order to completely fulfill the confocal condition, the entrance pinhole should be not greater than 25 μm^30^. Conversely, we chose a pinhole size of 70 μm to increase the collected intensity to ensure an excellent spectrum quality with an acquisition time compatible with cell viability.

### Cell culture, oncogene expression and sample environments

The NIH/3T3 murine fibroblast cell line was purchased from the American type culture collection (ATCC). Cells were seeded onto silicon substrates that were sterilized using 70% ethanol and UV irradiation for 40 min. Cells were grown in Dulbecco’s modified eagle medium (DMEM) containing 10% (v/v) heat-inactivated fetal bovine serum (FBS), 100 U ml^−1^ penicillin, 100 U ml^−1^ streptomycin (Sigma Aldrich, St Louis, MO, USA) and maintained at 37 °C in a 5% CO_2_ humidified atmosphere.

Some of the cells were transfected using Lipofectamine LTX with the expression vector pcDNA^TM^6/myc-His encoding the constitutively active mutant H-RasV12. The vector expressing the Ras mutant was previously described^31^. This mutation replaces the amino-acid glycine with a valine, which makes the GTPase constitutively GTP bound. Transfected fibroblasts were selected using 4 μg ml^−1^ Blasticidin-S for 5 days. The expression of H-RasV12 was assessed by immunoblotting detected by chemiluminescence using the ECL system (GE Biosciences). Cells were lysed at 4 °C in RIPA buffer (50 mM Tris-HCl pH 8, 150 mM NaCl, 1% (v/v) NP-40, 0.1% (w/v) SDS, 0.5% (w/v) sodium deoxycholate) and proteins (30 μg for cell extract) were electrophoresed on SDS-PAGE and transferred to a PVDF membrane. The rabbit polyclonal anti-H-Ras antibody was from Santa Cruz Biotechnology (Santa Cruz, USA). As an internal control, the membrane was probed with mouse monoclonal anti-β-actin (Sigma-Aldrich, St Louis, MO, USA). Donkey anti-rabbit and sheep anti-mouse HRP-linked secondary antibodies (GE Biosciences, Piscataway, USA) were used according to the manufacturer’s instructions.

For the fluorescence microscopy analysis, fibroblasts were seeded onto glass cover slips. After 24 h, cells were washed three times with phosphate-buffered saline (PBS) and fixed with 3.7% formaldehyde/PBS for 15 min at RT. Cover slips were rinsed three times with PBS, permeabilized with 0.1% Triton X-100/PBS for 15 min at RT, blocked with 5% FBS/0.1% Triton X-100/PBS for 30 min at RT and then incubated with 1 unit ml^−1^ of Alexa Fluor 488 Phalloidin (Thermo Fisher Scientific, Waltham, MA, USA) for 30 min at RT. Cells were then washed with PBS and mounted on glass slides using Vectashield with DAPI (Vector Laboratories Inc, Burlingame, CA, USA). Fluorescence microscopy analysis was performed using a Nikon TE2000 microscope with a 60 × objective.

For the spectroscopic measurements, the cells were washed twice in phosphate-buffered saline (PBS), and for all experiments the substrate was immersed in a Petri dish containing HEPES-buffered standard physiological solution (NaCl 136.4 mM, MgCl_2_ 0.53 mM, KCl 5.4 mM, CaCl_2_ 1.8 mM, glucose 5.5 mM and HEPES 5.5 mM pH 7.4) to ensure an appropriate volume of buffer and optimal pH conditions for the cells during the measurement session. The thermalization at 37 °C was guaranteed by a temperature controlled copper cell surrounding the Petri dish. The sample environment was assembled on a translation stage (a PI 611-3S Nanocube XYZ closed loop) which reached a resolution of 10 nm in a motion range of 100 μm for each axis thanks to the piezoelectric control. This allowed the observation of single live cells using a water-immersion objective directly immersed in solution.

### Data analysis

#### *Raman microspectroscopy*

The data analysis was performed using in-house software that eliminates spikes from the affected spectra, subtracts the weak luminescence background using a sp-line algorithm and subtracts the buffer signals from the spectra. It should be noted that the shape of Raman spectra changes by analyzing different points of a chemically heterogeneous system. In particular, if the frequency position of any given peak is the signature of a chemical bond of a specific molecule, the intensity of the Raman peak is proportional to the concentration of that species multiplied by its optical activity. Therefore, Raman spectra are able both to probe the relative changes in the concentration of the same species in different points and to follow the changes in the relative concentrations between two different species.

#### *Brillouin light scattering*

**Figure 2:**
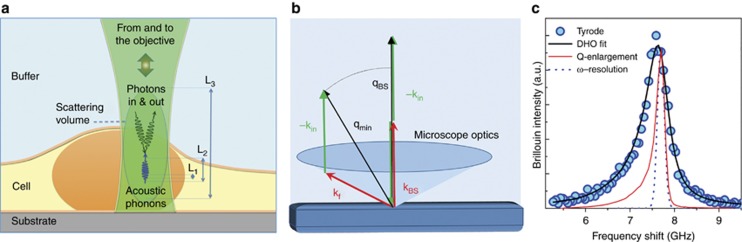
(**a**) Schematic micro-Brillouin scattering diagram. Incoming light interacts with acoustic phonons within the scattering volume to yield the Brillouin scattering effect. L_1_, L_2_ and L_3_ denote the relevant length scales for this interaction, given by acoustic wavelength, acoustic attenuation and scattering volume, respectively. (**b**) Sketch of the collection geometry. Because of the high NA of the optics, the exchanged wavevector **q** has a significant spread. The maximum value |**q**_BS_| corresponds to the back-scattering condition while the minimum value (|**q**_min_|~0.85|**q**_BS_|, for NA=1.2) corresponds to photons collected by the external region of the microscope lens. (**c**) Tyrode spectrum (cyan dots) compared with the spectrometer ω-resolution (blue dashed line) as measured from the elastically scattered light and with the optics induced q- enlargement (red line). The q-enlargement was measured on a sample with negligible intrinsic linewidth (a SiO_2_ slab) and rescaled to the Tyrode frequency by the sound velocities ratio. The reported fit (black line) takes into account the light scattered at different wave-vectors according to their measured weight (Equation (2) in the text).

The three main spatial scales involved in the Brillouin scattering experiments are outlined in Figure 2a. They are provided by the wavelength and attenuation of the acoustic modes responsible for the scattering of green laser light focused and collected by the microscope objective within the finite scattering volume. The smallest spatial scale is L_1_ ~0.1 μm, which is the wavelength of acoustic modes responsible for the scattering process; the intermediate scale is L_2_ ~1 μm, which is the attenuation length of acoustic modes; the largest scale is L_3_ ~1–10 μm, which is the size of the scattering volume. From this simple scheme, it is apparent that the spatial resolution in Brillouin mapping is limited by L_2_ since, even in the case of a more stringent confocal condition for the optical microscope, acoustic modes would make an average of the mechanical properties over such a distance.

For inhomogeneous samples, a condition quite common in biological matter, mechanical inhomogeneity on a length scale much smaller than L_1_ is hidden by the acoustic field and an effective homogeneous medium is revealed with average elastic constants.

Conversely, inhomogeneity at a length scale approaching L_1_/10 or larger is the most challenging, since acoustic scattering effects can become important and give rise to anomalous dispersion and attenuation of acoustic modes (see, for instance, Refs. 32, 33).

Finally, for samples which are inhomogeneous at length scales larger than L_2_, an inhomogeneous broadening of Brillouin peaks can be expected, which can be treated as described as follows. A more detailed discussion on the effect of spatial and temporal inhomogeneity in the sample on the results of Brillouin scattering from biological matter can be found in Ref. 34.

Within a homogeneous portion of the sample, that is, for inhomogeneity much smaller than L_1_, the scattered light is shifted in frequency by an amount that is proportional to *V*, the velocity of the acoustic modes through the relationship *ω*_B_=2 *π ν*_B_=±*V*·q, where **q**=n (**k**_f_−**k**_i_) is the momentum exchanged in the scattering process, n is the refractive index of the sample and **k**_i_ (**k**_f_) is the wavevector of incident (scattered) light. Longitudinal sound velocity is directly related to the longitudinal elastic modulus *M*=*ρ* *V*^2^.

In case of disordered materials, damping mechanisms give rise to attenuation of acoustic modes, and the spectrum can be described by a damped harmonic oscillator (DHO) which presents peaks of ~2*Γ*_B_ width:


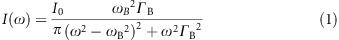


Note that similar to the peak position, the linewidth *Γ*_B_ is also not an intrinsic property of the material but is rather dependent on the exchanged wavevector. The kinematic viscosity of the sample, *D,* can be obtained by the relationship *D*=*Γ*_B_/q^2^ Ref. (35, 36).

Apart from its intrinsic width due to the attenuation of acoustic waves, the broadening of the Brillouin peak has two spurious sources: (i) the finite resolution of the spectrometer, which can be estimated from the elastic scattering peak, and (ii) the range of the exchanged wave vectors, Δq, collected by the finite acceptance angle of the detection optics, which reflects the frequency of the scattered light. In contrast to the usual macro Brillouin setups, the use of a high numerical aperture (NA) microscope objective for the focusing of the laser and collection of the scattered radiation induces a relevant q indeterminacy in a backscattering configuration too^37^ as shown in Figure 2b. As an example, for a standard 50 × objective, the NA in air is ~0.6, which translates into a Δq/q_BS_ ~5%, q_BS_ as a backscattered wavevector. This is even more relevant for the 60 × immersion objective in our setup where Δq/q_BS_ is greater than 10%.

The *a priori* evaluation of the broadening would require knowledge of the actual response of the objective and the spectrometer optics. This is usually a complex task. However, the collected intensity can be considered as a sort of convolution between the q-dependent spectrum and the q-dependent optical response. Therefore, measuring the spectrum of a material with a negligible intrinsic broadening, approximated to a Dirac delta function, allows for the direct evaluation of the whole response of the optical setup, R(q).

We followed such a procedure to characterize our setup using a vitreous silica sample immersed in distilled water as a reference (the data are shown in Figure 2c); a further correction was indeed required to take into account the refractive index mismatch between v-SiO_2_ and water.

The spectrum collected by the Brillouin spectrometer is therefore given by the integral:


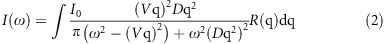


The resulting function was used to fit the collected data. The good fit quality obtained using Equation (2) is shown in Figure 2c for the buffer solution. As the scattering volume is occupied by more than one material whose characteristic size is greater than L_2_, the scattered intensity is calculated from the sum of the contributions of each component.

## Results and discussion

**Figure 3:**
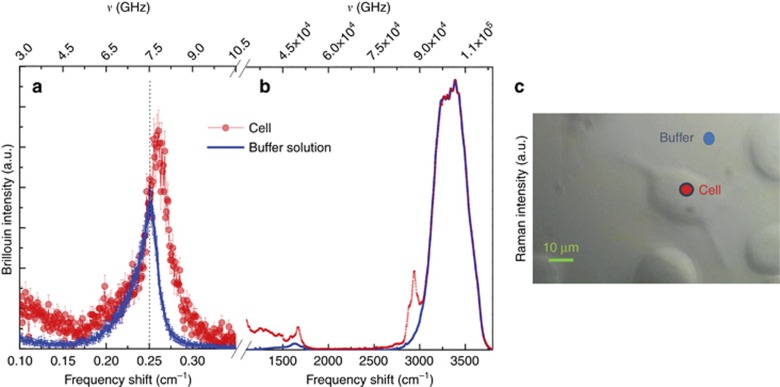
(**a**) Brillouin spectra (left) (**b**) and Raman spectra (right) acquired by focalizing inside the cell (red dots) and in the buffer solution (blue line). The acquisition time for the whole spectrum is 300 s. The Raman spectra show the characteristic peaks of the local biochemical constituents of the cell: the lower frequency region, below 1650 cm^−1^, is the ‘fingerprint’ region where the characteristic peaks of any cellular compound are present. The CH band in the high-frequency region ~3000 cm^−1^ is composed of different contributions: lipids (centered at ~2855 cm^−1^ and 2880 cm^−1^)^38, 39^ and proteins (centered at ~2935 cm^−1^)^39, 40^. The important contribution of the water vibrational modes (centered at approximately 1600 cm^−1^ and 3400 cm^−1^) is mainly due to the presence of the buffer solution inside the scattering volume (as shown in Figure 2a) as well as the hydration water, which is abundant in the total cellular composition. (**c**) Microscope image of the investigated sample with the two measured points evidenced by blue and red dots.

Typical raw Brillouin and Raman spectra acquired by focalizing inside the cell are reported in Figure 3a and 3b, together with those acquired on the free substrate (red and blue spectra, respectively). The substrate spectrum is dominated by the buffer signals.

From the comparison of the raw data, we found that the Brillouin peak of the cell is located at a higher frequency with respect to the buffer. Furthermore, its spectral shape, in particular the width and the presence of a quasi-elastic component highlights the presence of a phonon damping processes^41^. This is the first key result of this study. The unprecedented contrast joined with the high-frequency resolution and the extreme statistical accuracy allow the detailed study of the shape of the Brillouin spectrum in a living cell: a micro-system rich in water immersed in a buffer environment.

**Figure 4:**
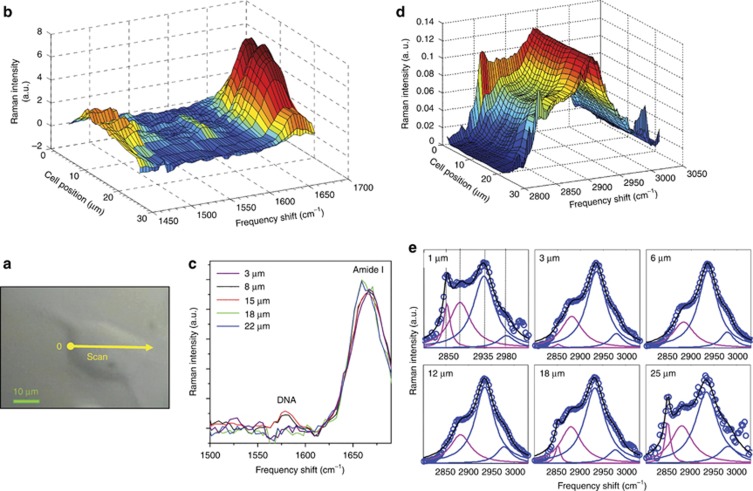
(**a**) Cell image. The yellow point is the initial investigated point of the measurements. The straight arrow along the cell shows the direction in which the measurements were performed while the position in the spectra are the distance from the starting point. Raman spectra (**b** and **c**) in the low (1400–1700 cm^−1^) and (**d** and **e**) the high (2800–3050 cm^−1^) frequency range as a function of the position inside the NIH/3T3 cell. (**e**) Deconvolution of the high-frequency carbon–hydrogen (CH) stretching band. The sum of four Lorentzian shape functions was used to fit the spectrum. The frequencies of the peaks were fixed to the main contributions assigned to different components (lipids 2850 cm^−1^ and 2880 cm^−1^; proteins 2935 cm^−1^, asymmetric stretch of methoxy 2975 cm^−1^)^38, 39, 40^, while their intensity and width were left as free parameters 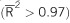.

**Figure 5:**
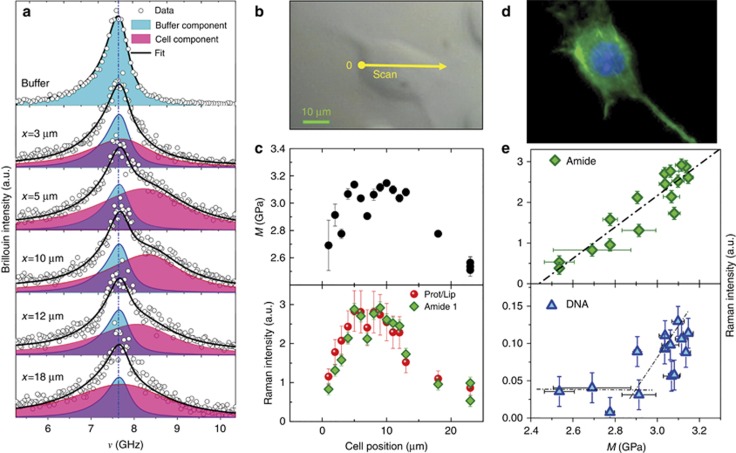
(**a**) Brillouin spectra and their deconvolution into cell and buffer components. The adjusted coefficient of determination 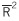 is always higher than 0.95 for the entire dataset. The fit was performed by considering a fixed shape of the buffer contribution (sound velocity and kinematic viscosity of the corresponding DHO function). Leaving these parameters as free, or even adding a further DHO function, would not lead to an increase in 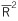. (**b**) Cell image. The yellow point is the initial investigated point for the illustrated measurements. The straight arrow along the cell shows the direction in which the measurements were performed. (**c**) Upper panel Longitudinal elastic modulus *M*=*ρ* *V*^2^ as a function of the position. In the fitting procedure, a cellular density *ρ*=1080 kg m^−3^ and a refractive index *n*=1.386 were assumed^13^; lower panel relative variation of the protein concentration as obtained by the area of the amide 1 Raman peak and by the deconvolution of the carbon–hydrogen (CH) stretching vibrational mode. (**d**) Fluorescence microscopy image of NIH/3T3 cell seeded onto glass cover slips. The average value of the nuclei size is (14±2) μm, in good agreement with the Raman spectroscopic signatures of the cell nucleus. (**e**) Raman peak intensities of the protein estimated by the intensity of the amide 1 peak and the DNA vs the longitudinal elastic modulus. A linear fit is shown in the upper panel as a dashed line (Pearson’s *r*>0.92) while the lines in the lower panel provide a visual representation.

The combined simultaneous Raman and Brillouin analysis allows a greater understanding of the investigated systems. Thanks to the high chemical specificity of the Raman spectroscopy and its sensitivity to the local environment, the distribution of the chemical species and their supramolecular structural arrangement can be correlated with the modulation of the biomechanical properties as we move across different cell compartments. The selected cells are adherent murine fibroblasts that mainly develop in the *x*–*y* plan. To assess the spectroscopic ability to monitor biomechanical and biochemical modifications in a single living cell, we performed a linear scan crossing the cell as shown in Figures 4 and 5 and acquired Brillouin and Raman spectra.

From the Raman spectra, markers of different chemical species can be obtained by analyzing the frequency region below 1650 cm^−1^, termed the fingerprint region, as well as the high-frequency range, where the characteristic band of the CH_2_ and CH_3_ stretching vibrations are located (at ~2950 cm^−1^). Raman imaging of selected spectral regions are shown in Figure 4b and 4d, as a function of the position inside the cell. The changes in the intensity of the peaks intensities, evidenced by the pseudo-colors in the maps, highlight the different concentrations of chemical species and their distribution in the cellular regions.

In Figure 4b, once the buffer solution was removed, the variations in the DNA peak centered at approximately 1580 cm^−1^ and the amide 1 peak centered at ~1650 cm^−1^ are easily visible. The former appears only in the central part of the cell in a region whose size is comparable to the size of the nuclei as measured by fluorescence microscopy. In the following, the DNA peak will be used as the Raman spectroscopic marker of the nucleus position, while the amide 1 peak will be used as an evaluation of the protein concentrations.

In addition, the high-frequency band can be exploited to monitor the relative concentration of lipids and proteins^39^. The broad-band of the carbon–hydrogen (CH) stretching vibrational mode (2800–3050 cm^−1^) is composed of the overlap from multiple contributions, in particular, the CH_2_ stretching vibration primarily arising from lipids at ~2850 cm^−1^, and the CH_3_ stretching vibration primarily arising from proteins at ~2935 cm^−1^. Recently, by exploiting this frequency difference, the ability to separate protein-rich from lipid-rich regions in cells has been demonstrated. Using stimulated Raman scattering at two specific Raman shifts within the stretching vibrational mode, the map of protein and lipid distributions in cells and tissues has been obtained^40, 42, 43, 44, 45^. These experiments inspired our method: the main difference is that, instead of exploiting the resonance effect, we estimated the amount of a given species by a deconvolution procedure. As we move from the external part towards the inner part of the cell (Figure 4d and 4e), the low frequency side of the CH stretching profile assigned to the lipid component (2855 cm^−1^ and 2880 cm^−1^)^38, 39^ reduces its intensity with respect to the prominent band maximum assigned to the proteins^38, 39^ centered at ~2935 cm^−1^. In fact, while lipids are mainly localized in bio-membranes, proteins are present in many compartments and structures of the cell. The deconvolution procedure can be used to obtain indications of the relative variation of the protein and lipid components in the cells. In particular, the complex network that forms the cytoskeleton of the cell, which is mainly composed of crosslinked protein filaments that spread throughout the cytoplasm, will appear as a maximum in the intensity of the protein signals obtained from the low- and high-frequency regions.

Selected Brillouin spectra relative to the same positions investigated by Raman scattering are reported in Figure 5a. We moved from outside the cell (first spectrum referring to the buffer solution), then entered through the membrane, the cytoplasm, the nucleus and finally exited from the opposite side. From the raw data, clear modifications of the Brillouin line shape are visible and, especially for the spectra acquired in the central part of the cell, one can visually infer the simultaneous presence of two excitations. To obtain quantitative information on the frequency of the acoustic excitation and the viscoelasticity of different parts of the cell, data were fitted by the convolution of the measured resolution function R(q) with two DHO functions. The first peak can be safely assigned to the spectral contribution of the buffer component that is always present in the scattering volume. Only its intensity changes during the scan. As shown in Figure 5a, the results of the fit show that the frequency position of the second DHO changes while probing different cell parts, highlighting its sensitivity to the local cell elastic moduli. While the buffer contribution was easily subtracted from the raw Raman spectra, for the Brillouin spectra the careful data analysis performed herein was required in order to isolate the cell features. As shown, this procedure allowed the correct evaluation of the cellular biomechanics modulation, which, due to cellular morphology, is principally expected along the *x*–*y* plane.

Thanks to the deep characterization of the spectrometer response function and to the innovative analysis method, we report for the first time the accurate tracking of modifications of both the position and shape of the Brillouin peak in different cell positions, which are clearly distinguished from the contribution of the surrounding buffer medium.

The obtained longitudinal elastic modulus as a function of the position in the cell is reported in the upper panel of Figure 5c. A strong increase of ~20% in the elastic modulus occurs in the central region of the cell.

To correlate this evolution with the corresponding biochemical composition, we report the relative variation of selected Raman bands in the lower panel of the same figure. In particular, the area of the amide 1 peak and the proteins vs lipids ratio, which can both be considered as spectroscopic measures of the protein concentration, appear strictly correlated with the cell elasticity. Indeed, a linear dependence was observed (Figure 5e). This purely spectroscopic result confirms the key role of protein structures in conferring rigidity to the cell. Moreover, by analyzing the DNA signal vs. the elastic modulus, we found that the nucleus occupies the stiffer cell zone.

The ability of our technique to monitor biomechanical modifications was also tested by comparing the Brillouin data acquired in the control cells (NIH/3T3) with those obtained in the same cells after transfection (H-RASV12). In fact, NIH/3T3 are known to undergo oncogenic transformation upon the expression of constitutively active H-Ras by activating multiple downstream signaling pathways^46^.

**Figure 6:**
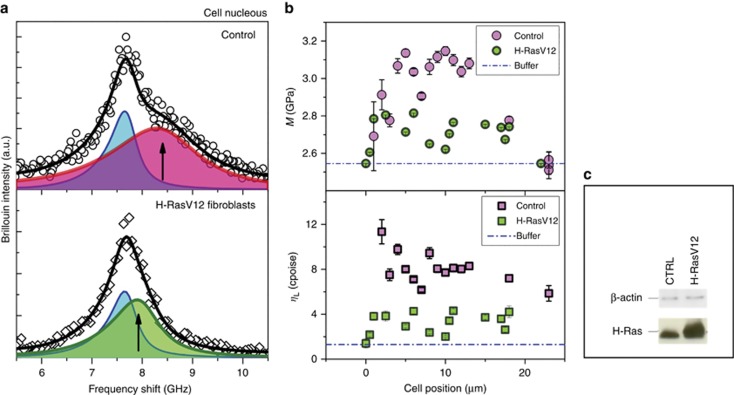
(**a**) Brillouin peak acquired in the nucleus of the control cell (CTRL NIH/3T3) in the upper panel and in transfected fibroblast (H-RASV12) in the lower panel. (**b**) Longitudinal elastic modulus *M*, *M*=*ρV*^2^ and longitudinal apparent viscosity *η*_L_=*ρ* D as a function of the position in the cell for the two cell conditions. (**c**) Cell extracts (30 μg) were separated by SDS-PAGE, electrotransferred and probed with anti-H-RasV12 antibody. β-actin was used as a control marker.

Figure 6a shows the comparison between the Brillouin spectra acquired in the two cases while probing the nucleus of the cell. A clear decrease in the peak frequency accompanied with a decrease in its width was measured in cells expressing the H-RasV12 oncogene. The biomechanical changes are quite visible along the whole cell dimension revealing that the transfected cells present a generally lower longitudinal apparent viscosity and lower elastic moduli values with respect to healthy cells (Figure 6b). These properties are in agreement with the invasive potential observed in cancer cells; their increase in deformability enhances their squeezing ability through narrow spaces of the extracellular matrix, favoring *in vivo* dissemination and metastasis.

Aberrations in nuclear morphology, including size, shape^47^ and stiffness^48^, are documented in the literature and have been assigned to the deregulation of protein expression in several cancer types. However, the contribution of aberrant regulation in cancer progression remains unclear, and the study of its correlation with biophysical parameters, such as nuclear stiffness and deformability, appears to be of great importance. Our findings indicate that the stiffness of the nucleus and its eventual mechanical integration into the cytoskeleton through nuclear lamins are altered in cancer cells as observed by the overall reduction in cell elastic moduli and apparent viscosity.

The softening of cancer cells with respect to healthy cells has already been observed in the literature^49, 50^, even suggesting that this property could be a new bio-marker for cancer diagnosis^51, 52, 53^. Our results significantly extend to the bulk, the previous analysis performed by atomic force microscope (AFM), which probed the system at lower frequencies and primarily on the surface^54^. In this regard, it is worth noting that the viscoelastic nature of biological materials is responsible for the order-of-magnitude increase in the elastic moduli measured in the high-frequency regime by Brillouin scattering. This evidence is explained and formally justified in the framework of generalized hydrodynamics, which introduces a complex frequency dependent elastic modulus *M*(*ω*)^41^. Recent Brillouin characterization of tissues and cells (see, for instance, Refs. 11, 55) verified the existence of a scaling law between the elastic moduli in the GPa range measured by Brillouin and those in the kPa range measured by quasi-static techniques, proving that BLS is able to properly visualize the existence of elastic heterogeneities during the mapping of biological matter. Moreover, as a non-invasive and contactless technique, the applicability of the microspectroscopy Brillouin analysis can be extended in the future to *in vivo* analysis.

## Conclusions

The present work represents a proof-of-concept investigation to test the capabilities of the joint Raman-Brillouin microspectroscopy approach for key applications in the field of cellular biology. This novel technique covers a wide frequency range, from a fraction of a GHz to hundreds of THz, and, through a multiscale analysis, provides access to the collective vibrational properties of the cell as well as its molecular modes. As a multispectral technique, it represents a unique tool to reveal the biomechanical modulation of subcellular compartments correlated with their biochemical composition in a contactless manner, i.e., without indentation while still probing the sample in depth. The accurate study of the Brillouin line shape proposed herein provides a complete and deep characterization of the viscoelastic properties of living cells, offering a new method with respect to force microscopy techniques. In fact, although it possesses higher spatial resolution, AFM is unable to probe the chemical composition and internal elasticity of cells.

From a biological perspective, the role played by the cytoskeleton in the effective elasticity of the cell is obvious from this study. The considerable reduction in both the elastic modulus and apparent viscosity measured in living cells after oncogene expression can therefore be explained by a general modification of the cytoskeletal properties assigned to the deregulation of protein expression. In this regard, the invasive potential of cancer cells is clearly correlated to the modification of their mechanical properties, favoring their diffusion into tissues.

The ability of this technique to follow the spatial and eventual temporal modulations of cellular mechanical properties, which are intimately related to physiological or pathological processes, could have a disruptive impact on future biomedical applications.

## Author contributions

SC initiated the project and provided guidance. DF designed the instrument development. SM performed Raman and Brillouin measurements. MM performed the Brillouin data analysis. SC performed the Raman data analysis. LU and KS provided biological materials, performed fluorescence microscopy and immunoblotting study, and, together with CE and MdS, provided guidance on the biological interpretation of experimental data. SC and MM wrote the manuscript with contributions from all authors.
